# PRPF6 promotes metastasis and paclitaxel resistance of ovarian cancer via SNHG16/CEBPB/GATA3 axis

**DOI:** 10.32604/or.2022.03561

**Published:** 2022-08-31

**Authors:** HAN WANG, YINGYING ZHOU, SIYANG ZHANG, YA QI, MIN WANG

**Affiliations:** Department of Gynecology and Obstetrics, Shengjing Hospital of China Medical University, Shenyang, China

**Keywords:** Ovarian cancer, PRPF6, SNHG16, Paclitaxel resistance, GATA3

## Abstract

Metastasis and paclitaxel (PTX) resistance are the main reason for the poor prognosis of ovarian cancer (OC). Evidence showed that RNA-binding proteins (RBPs) and long noncoding RNAs (lncRNAs) can modulate post-transcriptional regulation. The aim of this study was to determine the relationship among RBP, lncRNA and OC and to further guide clinical therapy. Immunohistochemistry revealed that pre-mRNA processing factor 6 (PRPF6) was upregulated in OC chemoresistant tissues and was closely related to advanced (Federation of International of Gynecologists and Obstetricians) FIGO stages and chemo-resistance. PRPF6 promoted progression, and PTX resistance *in vitro* and *in vivo*. And the transcripts of small nucleolar RNA host gene SNHG16-L/S were differentially expressed in OC cells and tissues as detected through real-time PCR (RT-PCR). SNHG16-L/S had opposite effects on progression and PTX resistance in OC. Mechanistically, SNHG16-L inhibited GATA-binding protein 3 (GATA3) transcription by binding to CCAAT/enhancer-binding protein B (CEBPB). Moreover, PRPF6 induced the alternative splicing of SNHG16, causing downregulation of SNHG16-L and, leading to the upregulation of GATA3 expression to further promote metastasis and PTX-resistance in OC. Totally, these data unveiled that PRPF6 promotes metastasis and PTX resistance of OC through SNHG16-L/CEBPB/GATA3 axis, which provides a new direction for OC treatment.

## Introduction

According to the global cancer statistics from the World Health Organization, 313,959 new cases of ovarian cancer (OC) and 207,252 OC-related deaths were reported in 2020 worldwide [1,2]. Due to the lack of early symptoms and efficient screening methods, approximately 70% of patients reach an advanced stage at the time of diagnosis. The treatment of advanced OC involves surgery with platinum and paclitaxel (PTX) chemotherapy [3]. The high rate of recurrence and metastasis, and chemoresistance in patients with OC leads to a poor prognosis. The potential pathological mechanisms of PTX resistance in OC may be very complicated, and current research has not reached a consensus. It is generally accepted that the mutation of β-tubulin and interference with the polymerization of microtubules are the prominent mechanisms of PTX resistance in OC [4]. PTX is a mitotic inhibitor, promotes the polymerization of tubulin, and blocks its depolymerization into subunits, inducing cell cycle arrest and thus plays an anti-tumor effect [5]. Many studies have confirmed that a high expression of β-tubulin III can reduce tubulin assembly and, decrease the binding rate of tumor cells to PTX [6]. Despite advances in OC diagnosis and treatment, the main obstacles to cure are still poor prognosis and high recurrence rates. Therefore, it is essential to explore the key factors contributing to metastasis and PTX resistance in OC, and to seek novel strategies for the treatment of OC patients.

Long non-coding RNA (lncRNAs) are a class of non-coding RNAs with a length of more than 200 nucleotides, and can participate in various cancers [7–11]. Small nucleolar RNA host gene 16 (SNHG16) is located in chromosome 17q25.1, has been shown to be involved in the mechanisms of various cancers. Studies have shown that SNHG16 can competitively combine with a variety of miRNAs to influence the expression of its target genes and promote the proliferation, invasion, and migration of various tumor cells such as nasopharyngeal carcinoma, breast cancer, cervical cancer, esophageal cancer and osteosarcoma [12–16]. However, the molecular mechanism of SNHG16 in OC is still in its early stage of development.

RNA-binding proteins (RBPs) can bind to RNA to further involved in RNA alternative splicing (AS), transcription and translation [17]. AS refers to the splicing of pre-mRNA to produce different transcripts [18]. Pre-mRNA processing factor 6 (PRPF6) is one of the components of small nuclear ribonucleoprotein (snRNP) and plays an important role in AS [19]. Mutations in C-terminal TPR domain of PRPF6 lead to synthesis disorders of the U4/U5/U6 snRNP complex and promote the occurrence of autosomal dominant retinitis pigmentosa [20]. PPRF6 can interact with targeted RNA, increase the expression of targeted transcripts with oncogenic function, and promote tumor proliferation and metastasis. For example, PPRF6 regulates the AS of the oncogenic transcript of ZAK to drive proliferation and metastasis in colon cancer [21]. PRPF6 activates AR/AR-Vs to promote the progression of hepatocellular cancer and prostate cancer [22,23]. Our preliminary research found that PRPF6 may be a target gene of miR-134 mediating the regulation of PTX resistance in OC [24]. However, its specific function and mechanism in OC have not been studied.

In our study, we found that two SNHG16 transcripts, which are defined as SNHG16-L (SNHG16-001, ENST00000448136.5) and SNHG 16-S (SNHG16-002, ENST00000590435.5), were differentially expressed in PTX-sensitive OC cell line-SKOV3 and PTX-resistant OC cell line-SKOV3-TR30. And SNHG16-L/S were differential expressed in chemosensitive and chemoresistant OC tissues. Further functional experiments demonstrated the opposite effects of SNHG16-L/S and PRPF6 on progression and PTX resistance in OC. SNHG16-L could bind to CEBPB to further inhibit transcriptional activity of GATA3. In addition, PRPF6 was highly expressed in chemoresistant tissues and was closely related to the advanced FIGO stages. PRPF6 induced the AS of SNHG16 to upregulate GATA3 expression to promote metastasis and PTX resistance. This study may help in finding a valuable target for OC therapy.

## Materials and Methods

### Patients and tissues

Paraffin sections of OC tissue samples from 68 patients were collected and OC tissue samples for Real-Time PCR from 50 patients were obtained from the department of Gynecology and Obstetrics of Shengjing Hospital of China Medical University from 2018–2020. At least two pathology experts jointly determined the postoperative pathology of in all the cases. Of these, 31 chemoresistant cases and 37 chemosensitive cases were determined according to NCCN guidelines. All patients have provided signed the informed consent and the experimental protocol was approved by the Institutional Medical Research Ethics Committee of the Shengjing Hospital of China Medical University (2020PS274K-X1).

### Cell culture

The PTX-sensitive OC cell line, SKOV3, was acquired from the Tumor Cell Bank of the Chinese Academy of Medical Sciences (Beijing, China). The PTX-resistant OC cell line, SKOV3-TR30, was derived from SKOV3 and provided by Zhejiang University affiliated Obstetrics and Gynecology Hospital (Hangzhou, China) [25]. All the cells were cultured in RPMI/1640 (Hyclone, USA) medium contains 10% fetal bovine serum (FBS, Procell, Wuhan, China) and 1% penicillin/streptomycin at 37°C with 5% CO_2_. SKOV3-TR30 cells were maintained with the addition of 20 nM of PTX (Sigma Aldrich, St. Louis Missourida, USA). 293T cells were cultured in DMEM (Hyclone, Utah, USA). Mycoplasma testing and short tandem repeats authentication has been carried out for all the cell lines used.

### Real-time PCR(RT-PCR)

Total RNA was isolated using Trizol reagent (Invitrogen, California, USA). cDNA was synthesized according to the manufacturer’s protocol (Takara, Japan). SYBR premix Ex TaqTM II (Takara, Japan) was used for PCR. The primers were synthesized by Sangong (Shanghai, China) and shown as follows: PRPF6: Forward: GAGGATGCTGACAGTTGTGTAG, Reverse: CCATGGTTCTTCTCGAAGTACG; SNHG16-L: Forward: CCAGTTACACAGGATGCCGTCTTG, Reverse: AGCTGATTGCCTTGGTGAGTCAAC; SNHG16-S: Forward: GCCAAGGTGAAGCGAGCTGAG, Reverse: GCAAGAGACTTCCTGAGGCACAT; CEBPB: Forward: GCACAGCGACGAGTACAAGA, Reverse: TGCTTGAACAAGTTCCGCAG; GATA3: Forward: GTCCTGTGCGAACTGTCAGA, Reverse: CGAGCTGTTCTTGGGGAAGT. The expression of RNAs was normalized by 2^−ΔΔCT^ method.

### Cell transfection

Cells were spread into 6-well plates with a density of 30%–50%. After culturing for 24 h, when cell fusion degree reaches 50%–70%, change the medium into serum-free medium. Add 2 ug plasmid DNA solution or 25 nM siRNA solution and 4 ul lipofectamine 3000 (Invitrogen, California, USA) transfection reagent into new tubes. After mixing evenly for 10 min. Transfer the mixed solution to the 6-well plate, and add serum-free medium to a final volume of 2 ml. After 4–6 h, change to serum-containing medium. siRNAs were synthesized from Ribobio (Guangzhou, China). The sequences were as follows: si-PRPF6-001: GAAGCGGGTTCTTCGGAAA, si-PRPF6-002: GGATCTAAATGACACCAAT, si-PRPF6-003: CTCGGAACCTTATCATGAA. The overexpression plasmids pHBLV-PRPF6, pcDNA3.1-SNHG16-L, pcDNA3.1-SNHG16-S, pcDNA3.1-CEBPB, and pHBLV-GATA3 were synthesized from Hanbio (Shanghai, China). Lipofectamine 3000 (Invitrogen, California, USA) was used transfection according to the instructions.

### Transwell assay

5 × 10^4^ cells were suspended in a serum-free medium and plated on upper transwell migration chambers (Corning Costar, California, USA). Transwell invasion assay was coated with Matrigel (BD, California, USA). The lower chambers added medium with 10% FBS. After cultured 24 h, the membranes were fixed with methanol and stained with 1% crystal violet. Five random fields (×400 magnifications) were counted and photographed under the light microscope.

### CCK8 assay

2000 cells were seeded in 96-well plate with 100 ul medium per well. We added 10 ul CCK8 reagent (Sigma, St. Louis Missourida, USA) at 0, 24, 48, 72, and 96 h. The optical density was measured at 450 nm. To test differing PTX sensitivities, we added various concentrations of PTX after seeded for 24 h. Then, added 10 ul CCk8 reagent at 48 h and examined in 2 h.

### Colony formation assay

Cells were plated in 6-well plates and incubated for 10 days (1000 cells/well). Then fixed with methanol and stained with 0.1% of crystal violet and calculated the number of visible colonies.

### Western blotting

Total protein was extracted via RIPA lysis (Beyotime, Shanghai, China) with phenyl-methane-sulfonyl fluoride and protease inhibitor. 10% SDS-PAGE gel electrophoresis with 30 μg protein per well was performed and then we transferred PVDF membranes. Antibodies PRPF6 (No. ab99292, 1:2000, Abcam, London, England), CEBPB (No. ab32358, 1:2000, Abcam, London, England), GATA3 (No. ab199428, 1:2000, Abcam, London, England), Vimentin (No. ab40497, 1:1000, Elabscience, Wuhan, China), E-cadherin (No. ab40285, 1:1000, Elabscience, Wuhan, China), N-cadherin (No. ab70061, 1:1000, Elabscience, Wuhan, China), β-tubulin III (No. YM0634, 1:2000, Immunoway, Wuhan, China), β-actin (No. AP0060, 1:5000, Bioworld, Wuhan, China) were incubated overnight at 4°C.

### RNA immunoprecipitation (RIP)

The experiment was conducted followed the manufacturer’s protocol of Magna RIP Kit (Millipore, Massachusetts, USA). The antibodies were PRPF6 (10 ug per reaction, Abcam, London, England) and CEBPB (10 ug per reaction, Abcam, London, England). RNA was extracted after detachment from the bead using protease K. The expression of SNHG16 pre-mRNA, SNHG16-L and SNHG16-S was determined using RT-qPCR.

### Chromatin immunoprecipitation (ChIP)

ChIP was performed according to the instruction of EZ ChIP Kit (Millipore, Massachusetts, USA). The antibody was CEBPB (10 ug, Abcam, London, England). The possible binding sites of CEBPB and GATA3 promoter were predicted via Jaspar, and specific primers were synthesized by Sangong (Shanghai, China), sequences were as follows: GATA3 promoter: Forward: CAAGCCCTTTGCCCCAT, Reverse: CAGGTAGAGTTTTCCCTTCACAA. The enrichment of GATA3 promoter was detected by RT-PCR.

### Dual-luciferase reporter assay

The luciferase plasmids pSI-Check2-GATA3 wild type/mutant type (wt-GATA3/mut-GATA3) were synthesized by Hanbio (Shanghai, China). According to the instruction of Dual-Luciferase® Reporter Assay System (Promega, Wisconsin, USA), the luciferase activity was detected.

### Immunohistochemistry (IHC)

The paraffin sections were deparaffinized and antigen retrieval was performed by adding citrate buffer (pH 6.1). The sections were then incubated with PRPF6 antibodies (1:250, Abcam, London, England) were diluted in 5% BSA, followed by DAB staining (Elabscience, Wuhan, China) and observation under microscope.

### Immunofluorescence (IF)

Cells growing on coverslips in 6-well plates were removed and fixed with 4% paraformaldehyde. Then, permeabilized in 0.5%–1.0% Triton X-100 for 10 min and blocked with 5% BSA for 30 min. The cells were then incubated with antibodies PRPF6 (1:150, Abcam, London, England), CEBPB (1:150, Abcam, London, England), GATA3 (1:150, Abcam, London, England) overnight at 4°C. Fluorescent-labelled secondary antibody (1:100, Proteintech, Wuhan, China) was added and incubated in the dark for 2 h. Cells were stained with DAPI and observed under the fluorescent microscope.

### Fluorescence in situ Hybridization (FISH)

Cells growing on coverslips in 6-well plates were removed. The SNHG16 probe were synthesized by Servicebio. The experiment was operated according to the protocol of Fluorescent *in situ* Hybridization Kit (Ribobio, Beijing, China). The slips observed under the fluorescence microscope.

### Xenografts in nude mice

The lentivirus containing siPRPF6 sequence was synthesized by Gene-Pharma (Shanghai, China). The SKOV3-TR30 cells were infected with lentivirus and obtained stably transfected cells. 4-weeks-old female BALB/cA-nu Mice (N = 3/group) were purchased from Huafukang (Beijing, China). The mice were randomly divided into four groups. The mice subcutaneously inoculated with cell suspension (200 µL, 5 × 10^6^ cells) into dorsal part to observe tumor growth. After 1 week, PTX (20 mg/kg) or saline was injected into tumor every 3 days for 3 weeks when tumor size reached 80–100 mm^3^. Animal experiments were performed according to the ethical guidelines for animal experiments and were approved by China Medical University Animal Welfare and Ethical Community (CMU2020341).

### Statistical analysis

The statistics were analyzed with SPSS 22.0. The results represented as the mean± standard deviation (SD). Data with normal distribution and homogeneity of variance were compared by paired sample *t*-test or non-paired *t*-test. One-way analysis of variance (ANOVA) was used for comparison among multiple groups. Repeated measures ANOVA, followed by the Bonferroni post hoc test, were used to analyze multiple groups at different time points. Significantly difference was set as *p* < 0.05 (*p < 0.05, **p < 0.01, ***p < 0.001).

## Results

### SNHG16-L/S had a different effect on the tumorigenesis, and PTX resistance in OC

**Figure 1:**
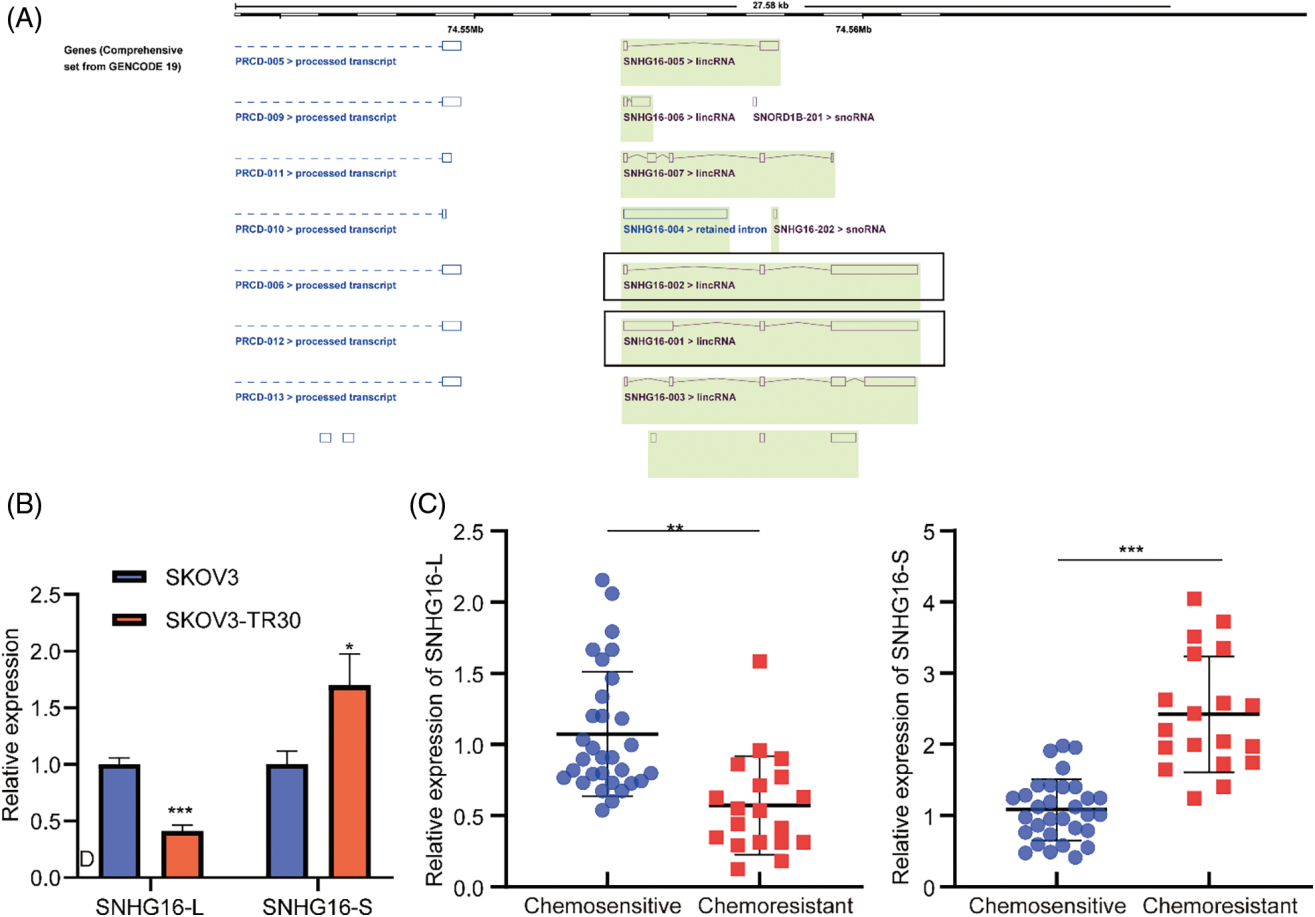
SNHG16-L/S had differential expression in OC cells and tissues. (A) Sequence differences of SNHG16-L/S compared based on ENSEMBL website. (B) RT-PCR was performed to analyze the expression of SNHG16-L/S in OC tissues. (C) RT-PCR was used to detect the expression of SNHG16-L/S in SKOV3 and SKOV3-TR30 cells. *p < 0.05, **p < 0.01, ***p < 0.001 (n = 3).

ENSEMBL annotation [26] showed that the difference in SNHG16-L/S was the existence of exon 1 (Fig. 1A). Through RT-PCR analysis, we found that the expression of SNHG16-L in the PTX-resistant cell line, SKOV3-TR30, was lower compared with that in the PTX-sensitive cell line, SKOV3. The expression of SNHG16-S was higher in SKOV3-TR30 (Fig. 1B). We then detected the expression in OC tissues. The results revealed that compared with chemosensitive tissues, SNHG16-L had lower expression in chemeresistant tissues while SNHG16-S had higher expression (Fig. 1C).

**Figure 2:**
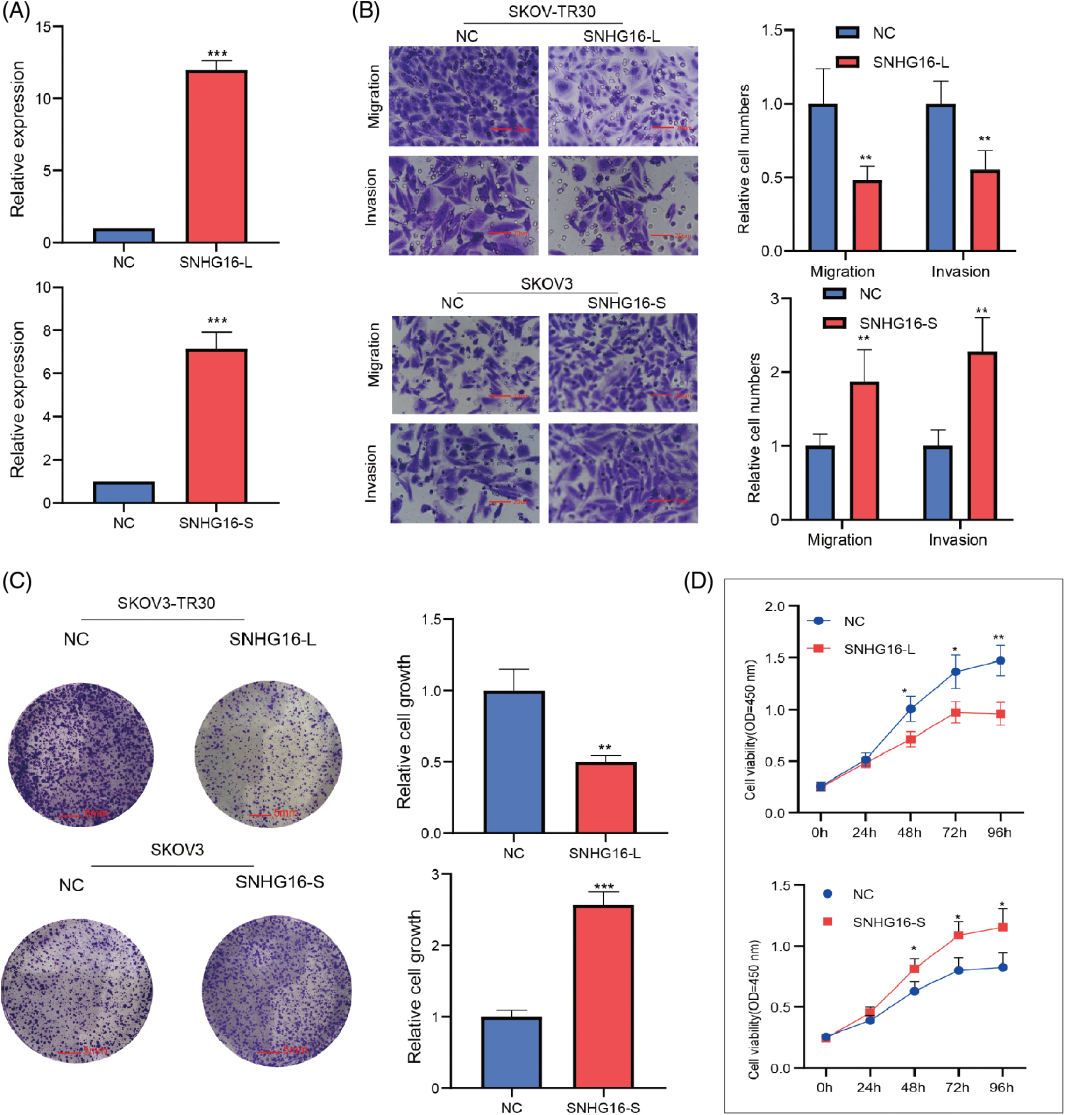
SNHG16-L/S had a different effect on cell migration, invasion and cell growth in OC. (A) Detection of expression of SNHG16-L/S after transfection of SNHG16-L vectors in SKOV3-TR30 and SNHG16-S vectors in SKOV3 through RT-PCR. (B) Cell migration and invasion were detected using transwell assays, (C) Cell colony proliferation was detected using colony formation experiment, (D) cell proliferation was detected using CCK8 assays. *p < 0.05, **p < 0.01, ***p < 0.001 (n = 3).

Then, full-length of SNHG16-L/S were cloned into specific vectors (pcDNA3.1) for overexpression studies. RT-PCR analysis confirmed that the SNHG16-L/S vectors significantly elevated SNHG16-L/S levels in SKOV3-TR30 and SKOV3 cells, respectively (Fig. 2A). Further functional experiments were performed to determine the effects of their expression levels. Transwell assays were performed to assess cell migration and invasion abilities. The results confirmed that the presence of OE-SNHG16-L decreased cell migration and invasion, whereas OE-SNHG16-S increased cell migration and invasion (Fig. 2B). We then used a colony formation assay to analyze cell growth and found that SNHG16-L overexpression inhibited cell growth, whereas SNHG16-S overexpression induced it (Fig. 2C). The results of the CCK8 assay also showed that overexpression of SNHG16-L and SNHG16-S had decreased and increased cell growth, respectively (Fig. 2D).

**Figure 3:**
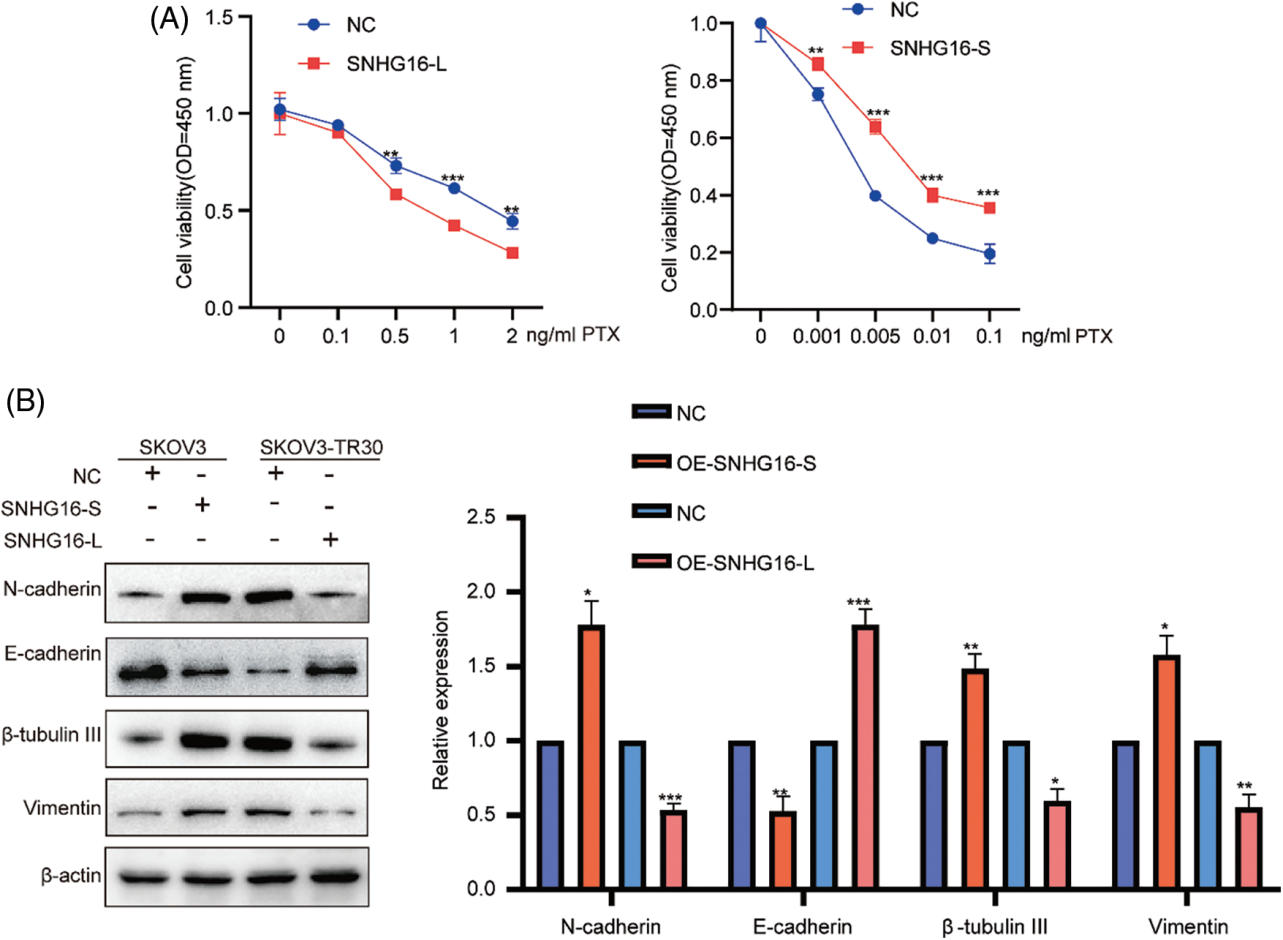
SNHG16-L/S had a different effect on PTX resistance and EMT in OC. After overexpressed SNHG16-L in SKOV3-TR30 and SNHG16-S in SKOV3 cells, (A) PTX resistance activity was detected using CCK8 vitality assays, (B) N-cadherin, β-tubulin III, vimentin and E-cadherin were detected by western blotting. *p < 0.05, **p < 0.01, ***p < 0.001 (n = 3).

Subsequently, SNHG16-L overexpression inhibited PTX-resistance, while SNHG16-S overexpression promoted PTX resistance in the CCK8 assay (Fig. 3A). In addition, western blotting was used to analyze the effects of SNHG16-L/S on the expression of (epithelial–mesenchymal transition) EMT-related proteins (N-cadherin, vimentin, and E-cadherin) and PTX resistance-related proteins (β-tubulin III). The results showed that overexpression of SNHG16-L decreased the expression of N-cadherin, β-tubulin III, and vimentin, and increased E-cadherin expression, while overexpression of SNHG16-S present the opposite trend (Fig. 3B).

### SNHG16-L bound to CEBPB and inhibited the transcriptional activity of GATA3 to further inhibit metastasis and PTX resistance of OC

**Figure 4:**
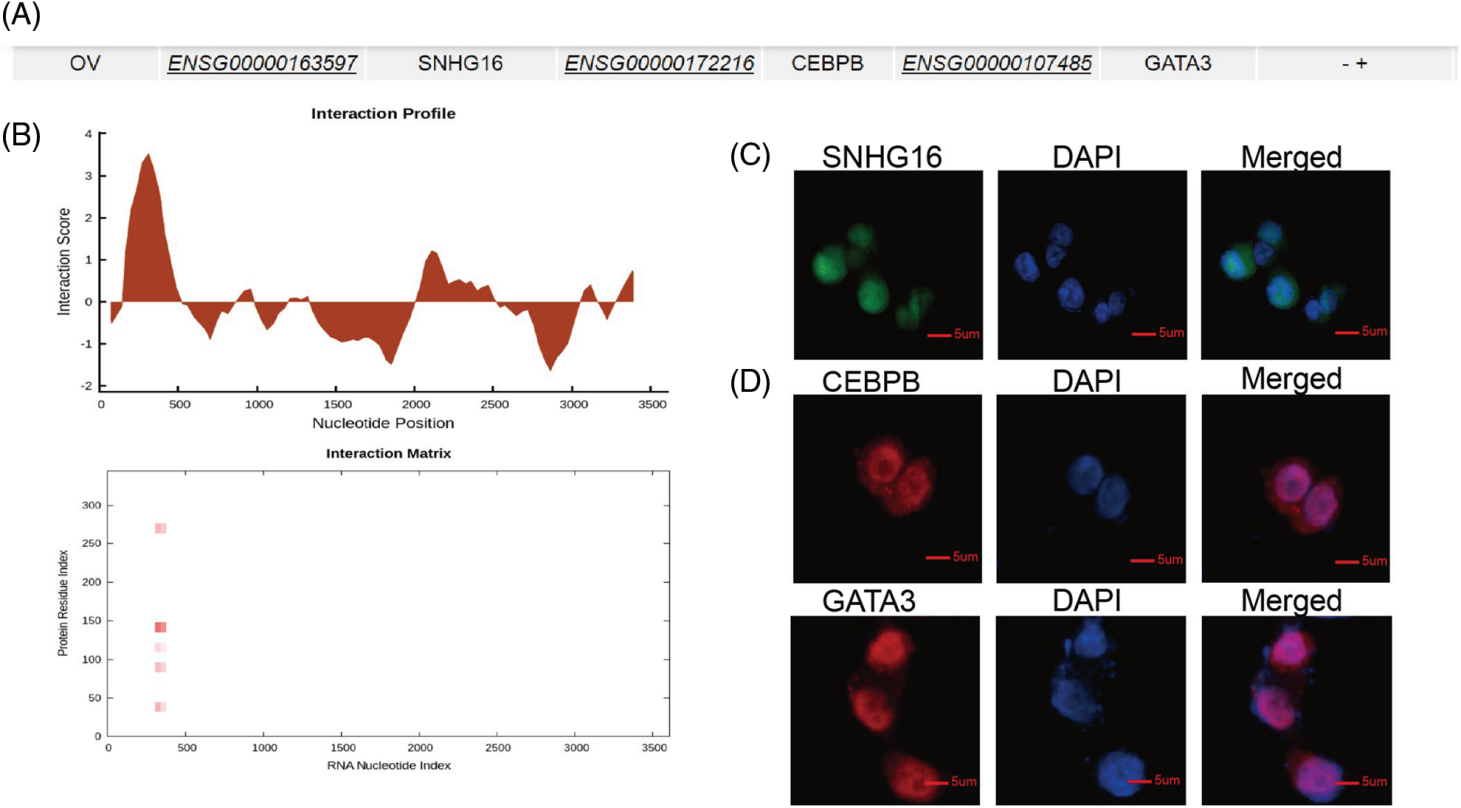
SNHG16 may be associated with CEBPB and GATA3. (A) A potential relationship among SNHG16, CEBPB and GATA3 was predicted through LncMAP. (B) CatRAPID omics predicted that CEBPB may bind to SNHG16. (C) FISH was used to detect the cellular location of SNHG16 in SKOV3-TR30 cells. (D) IF was used to detect the cellular location of CEBPB and GATA3 in SKOV3-TR30 cells.

LncRNAs can interact with DNA-binding protein to regulate transcription activity of target genes to mediate development of various cancer. Previous study shows that SNHG16 can combine with transcription factor SPI1 to upregulate PARP9 transcription to further promote tumorigenicity of cervical cancer [27]. To further explore the mechanism of SNHG16-L/S in OC, we used the LncMAP database (http://bio-bigdata.hrbmu.edu.cn/LncMAP) to find related transcription factors and targets genes. The results suggested that SNHG16 may regulate GATA3 via the transcriptional factor CEBPB in OC (Fig. 4A). We then predicted the binding sites of SNHG16 and CEBPB, and found that SNHG16-L combined with the CEBPB protein using CatRAPID-omics (http://service.tartaglialab.com/page/catrapid_omics_group) program (Fig. 4B). Then, we determined whether the cellular localization of SNHG16, CEBPB, and GATA3 was consistent. SNHG16 was located in the nucleus, as detected by FISH (Fig. 4C). Immunofluorescence analysis showed that CEBPB and GATA3 were expressed in the nucleus (Fig. 4D).

**Figure 5:**
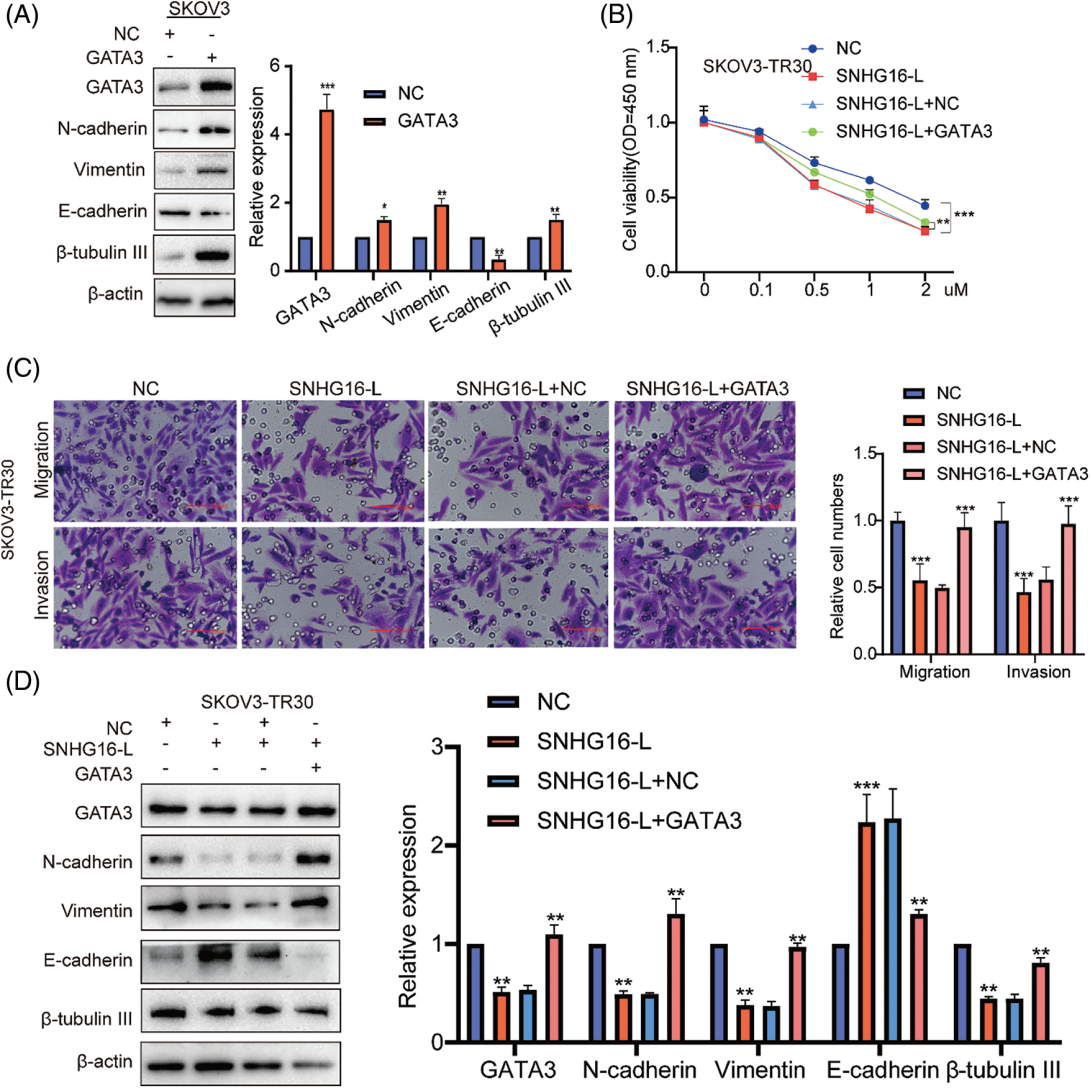
SNHG16-L inhibited GATA3 expression to further inhibit metastasis and PTX resistance of OC. (A) Overexpression GATA3 vectors in SKOV3 cells were used to construct GATA3 overexpressing cell lines and western blotting was used to examine the expression of N-cadherin, β-tubulin III, vimentin and E-cadherin. Overexpression of SNHG16-L and GATA3 in SKOV3-TR30 cells, (B) PTX resistance activity was detected by CCK8 vitality assays, (C) cell migration and invasion were detected by transwell assays, (D) western blotting was used to detect the expression of GATA3, N-cadherin, vimentin, β-tubulin III and E-cadherin. *p < 0.05, **p < 0.01, ***p < 0.001 (n = 3).

Previous studies have shown that GATA3 can be used as an independent risk factor to promote cell migration, invasion, and PTX resistance in OC [28,29]. We then overexpressed GATA3 in SKOV3 cells to examine the expression levels of N-cadherin, vimentin, β-tubulin III and E-cadherin using western blotting. The results showed that overexpression of GATA3 upregulated N-cadherin, vimentin, and β-tubulin III and downregulated E-cadherin (Fig. 5A). To determine whether SNHG16-L can regulate cell migration, invasion, EMT, and PTX resistance, we performed transwell, CCK8, and western blot assays after the overexpression of SNHG16-L and GATA3. These results indicated that SNHG16-L overexpression inhibited PTX resistance, migration, and invasion (Figs. 5B and 5C). It also downregulated GATA3, N-cadherin, vimentin and β-tubulin III, and upregulated E-cadherin expression. Overexpression of both SNHG16-L and GATA3 reversed the effects caused by the overexpression of SNHG16-L alone (Fig. 5D).

**Figure 6:**
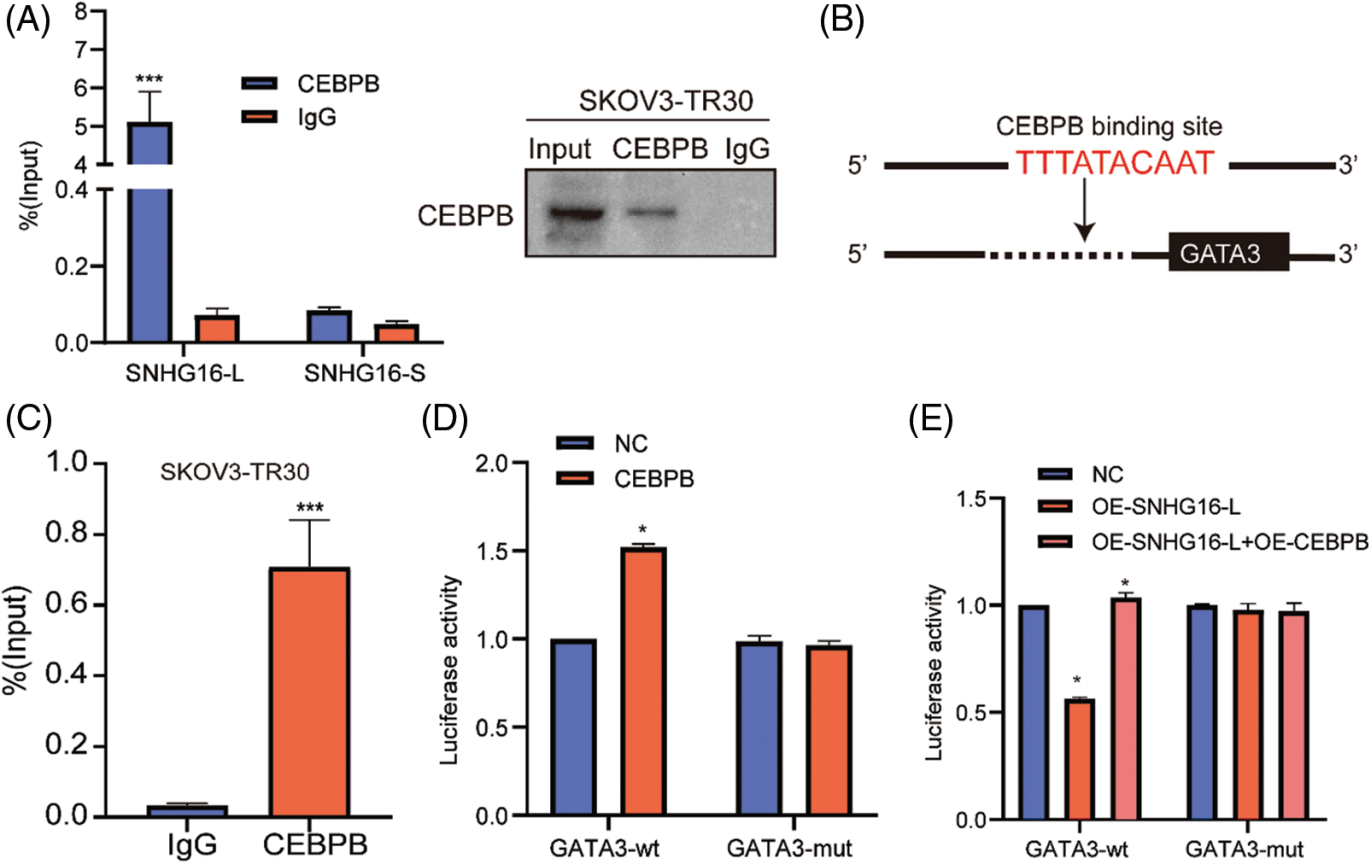
SNHG16-L could bind to CEBPB and inhibited the transcriptional activity GATA3. (A) RIP assay was carried out in SKOV3-TR30 cells with CEBPB antibody, the enrichment of SNHG16-L and SNHG16-S was detected by RT-PCR. (B) A schematic illustration of the proximal region between CEBPB and GATA3 promoter. (C) CHIP assay was performed in SKOV3-TR30 cells with the antibody of CEBPB, and the enrichment of GATA3 was detected by RT-PCR using specific primer of the predicted binding site of CEBPB and GATA3 promoter. (D, E) Dual-luciferase reporter assay was performed in 293T cells which were transfected with CEBPB vectors, SNHG16-L vectors, GATA3-WT and GATA3-MUT plasmids. *p < 0.05, **p < 0.01, ***p < 0.001 (n = 3).

To confirm the binding between SNHG16-L and CEBPB proteins, the RIP test was conducted in SKOV3-TR30 cells using the CEBPB antibody. Using RT-PCR, we found that CEBPB could bind to SNHG16-L but not to SNHG16-S (Fig. 6A). Furthermore, sequence analysis through Jaspar website (http://jaspar.genereg.net/analysis) revealed a potential binding site for CEBPB in the promoter region of GATA3 (Fig. 6B). ChIP detection using an antibody against CEBPB in SKOV3-TR30 was performed to explore whether CEBPB could bind to the GATA3 promoter. RT-PCR was conducted using specific primers of predicted sites, we found that CEBPB-bound complexes significantly enriched the 898-907 bp region of the GATA3 promoter (Fig. 6C). In addition, we constructed GATA3 luciferase mutant plasmids on this region and further validated our findings by using dual-luciferase reporter assay. The results showed overexpression of CEBPB enhanced the luciferase activity of the GATA3-wt group, but not the GATA3-mut group (Fig. 6D). This enhancement was weakened after the overexpression of SNHG16-L (Fig. 6E). Thus, SNHG16-L could downregulate the transcriptional activity of GATA3 via binding to CEBPB, further inhibiting cell migration, invasion, and PTX resistance of OC.

### PRPF6 was upregulated in chemo-resistant tissues and was related to the advanced FIGO stages of OC

**Figure 7:**
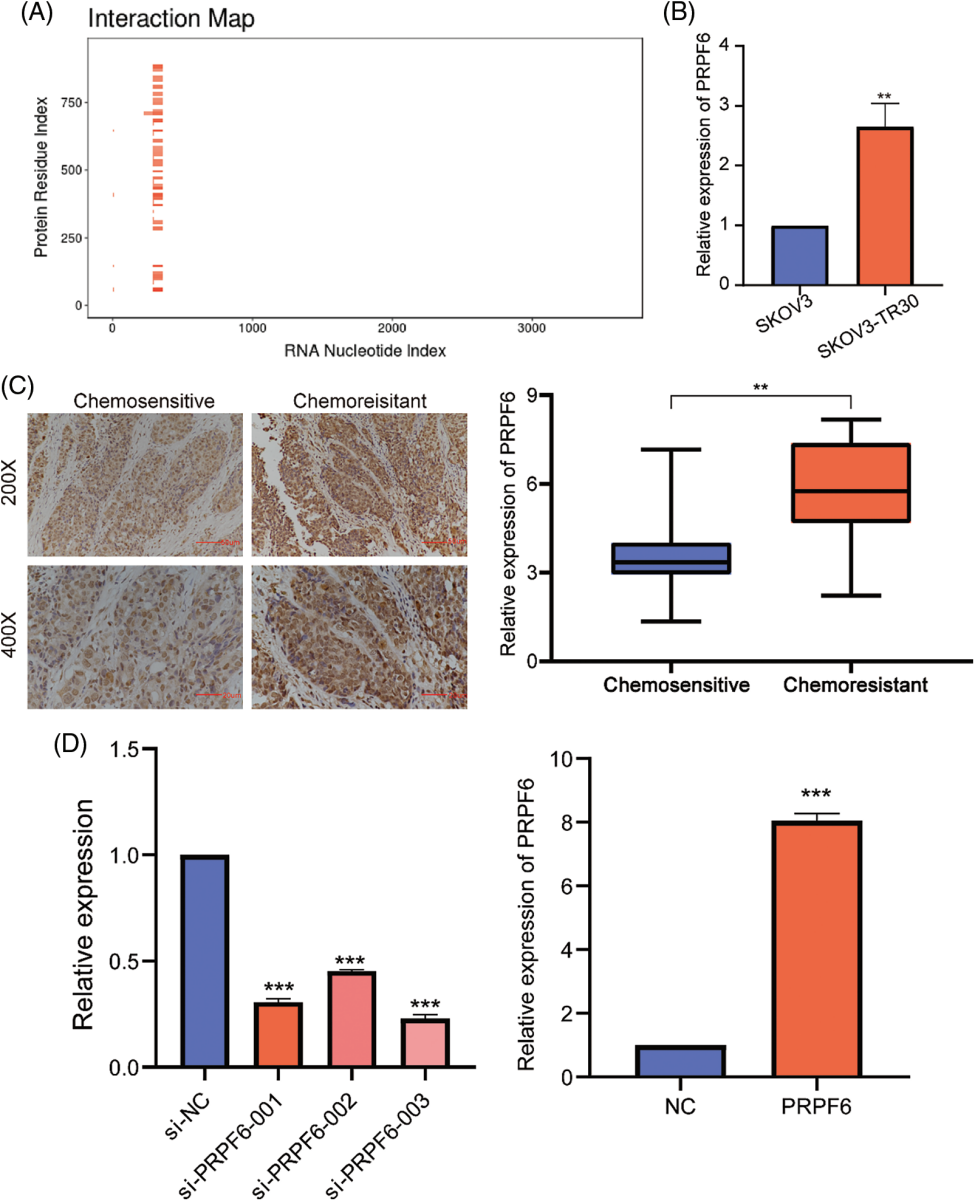
PRPF6 was upregulated in chemo-resistant tissues and was related to the advanced FIGO stages in OC. (A) CatRAPID omics predicted the putative binding sequence of SNHG16 pre-mRNA to PRPF6 protein. (B) RT-PCR was used to analyzed PRPF6 expression in SKOV3 and SKOV3-TR30 cells. (C) IHC was used to examine PRPF6 expression in OC tissues. (D) Three siRNAs were transfected in SKOV3-TR30 and overexpression vectors in SKOV3 to construct PRPF6 knockdown or overexpressing cell lines, as determined by RT-PCR. **p < 0.01, ***p < 0.001 (n = 3).

The differential function of SNHG16-L/S in OC indicated that SNHG16 may be mediated by AS. To find possible splicing factors binding to SNHG16, catRAPID omics analysis found that the 413–419 bp segment of the SNHG16 pre-mRNA exon 1 may be a potential binding site for PRPF6 (Fig. 7A). We used RT-PCR and IHC to detect the PRPFP6 expression in OC cells and tissues, respectively. We then analyzed PRPF6 expression in OC tissues using IHC and found that PRPF6 was located in the nucleus and was relatively higher in chemoresistant OC tissues (N = 31) than that in chemosensitive tissues (N = 37, Fig. 7B). We then used the median relative expression of PRPF6 in OC tissues, divided the patients into high-and low-expression groups, and analyzed the correlation between the expression level and clinicopathological characteristics of patients using the chi-square test. The results showed that PRPF6 was closely related to advanced FIGO stages but was not related to patient age, differentiation, or lymph node metastasis (Table 1). Furthermore, RT-PCR analysis revealed that PRPF6 was relatively higher in SKOV3-TR30 cells than that in SKOV3 cells (Fig. 7C). Three siRNAs were designed for knockdown studies and pc-DNA3.1-PRPF6 vector for overexpression studies. RT-PCR analysis showed that si-PRPF6-003 significantly downregulated PRPF6 in SKOV3-TR30 cells and was named si-PRPF6, and the vector markedly upregulated PRPF6 expression in SKOV3 cells (Fig. 7D).

**Table 1:** Correlation between PRPF6 and clinicopathological characteristics in OC patients

	**PRPF6**		
Variables	Low	High	*P-*value
Age			
<50	16	14	0.481
≥50	17	21	
FIGO			
I–II	21	10	0.004
III–IV	12	25	
Differentiation			
High	18	13	0.150
Moderate and low	15	22	
Lymph node metastasis			
Negative	18	14	0.230
Positive	15	21	
Chemo-sensitivity	28	9	0.000
Chemo-resistance	5	26	

### PRPF6 promoted tumorigenesis and PTX resistance in vitro and in vivo

**Figure 8:**
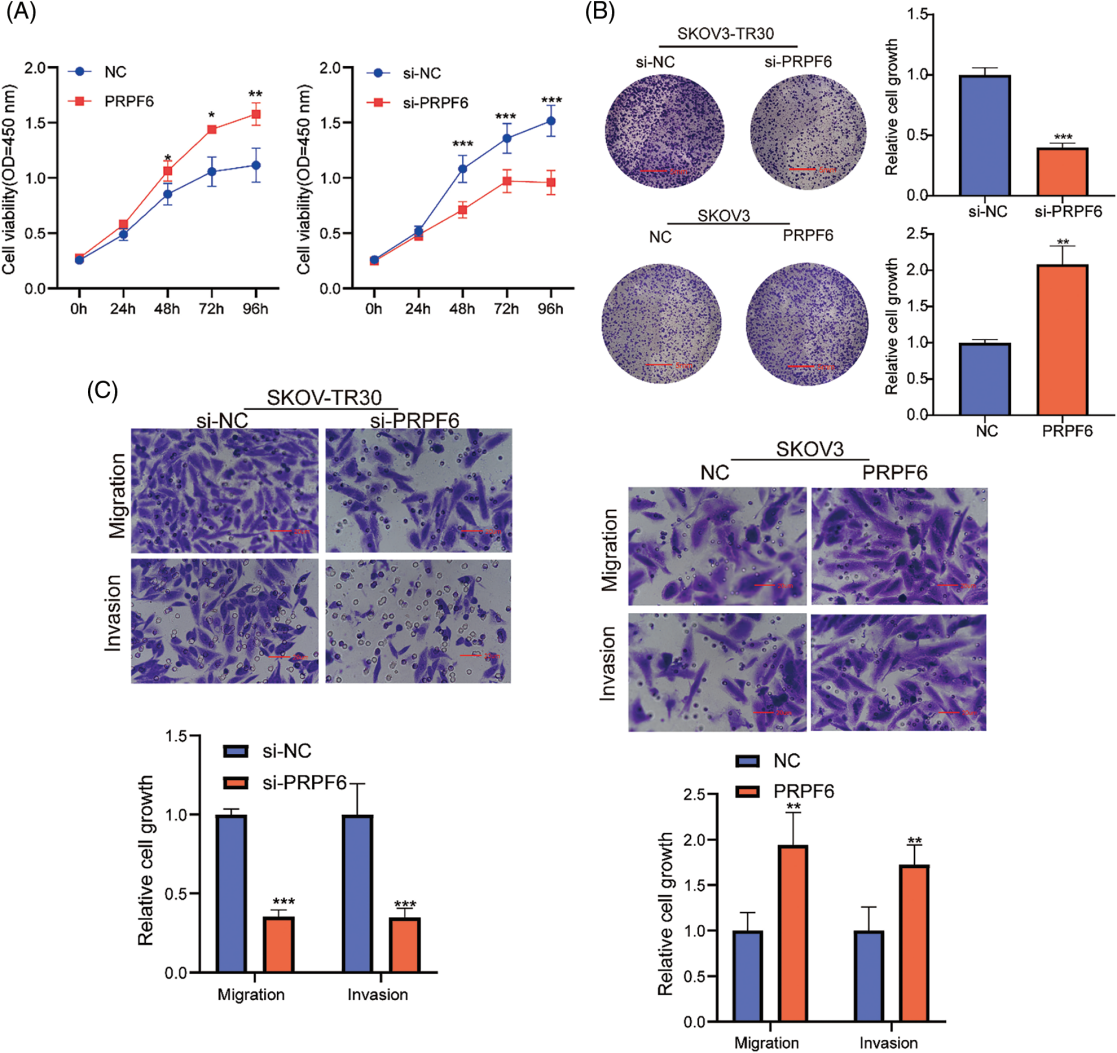
PRPF6 promoted cell growth, migration and invasion of OC. Silencing PRPF6 in SKOV3-TR30 and overexpressing PRPF6 in SKOV3, (A) Cell growth was analyzed through CCK8 assays, (B) cell colony proliferation was detected using colony formation experiment, (C), cell migration and invasion were detected by transwell assays. *p < 0.05, **p < 0.01, ***p < 0.001 (n = 3).

To identify the function of PRPF6 in OC cells, CCK8 assays indicated that si-PRPF6 and upregulation of PRPF6 significantly inhibited and promoted cell growth, respectively (Fig. 8A). The colony formation assay showed si-PRPF6 inhibited cell growth, whereas overexpression of PRPF6 promoted growth (Fig. 8B). Transwell assays were performed to assess cell migration and invasion. The results indicated that si-PRPF6 resulted in a decrease in invasion and migration, while PRPF6 overexpression resulted in an increase (Fig. 8C).

**Figure 9:**
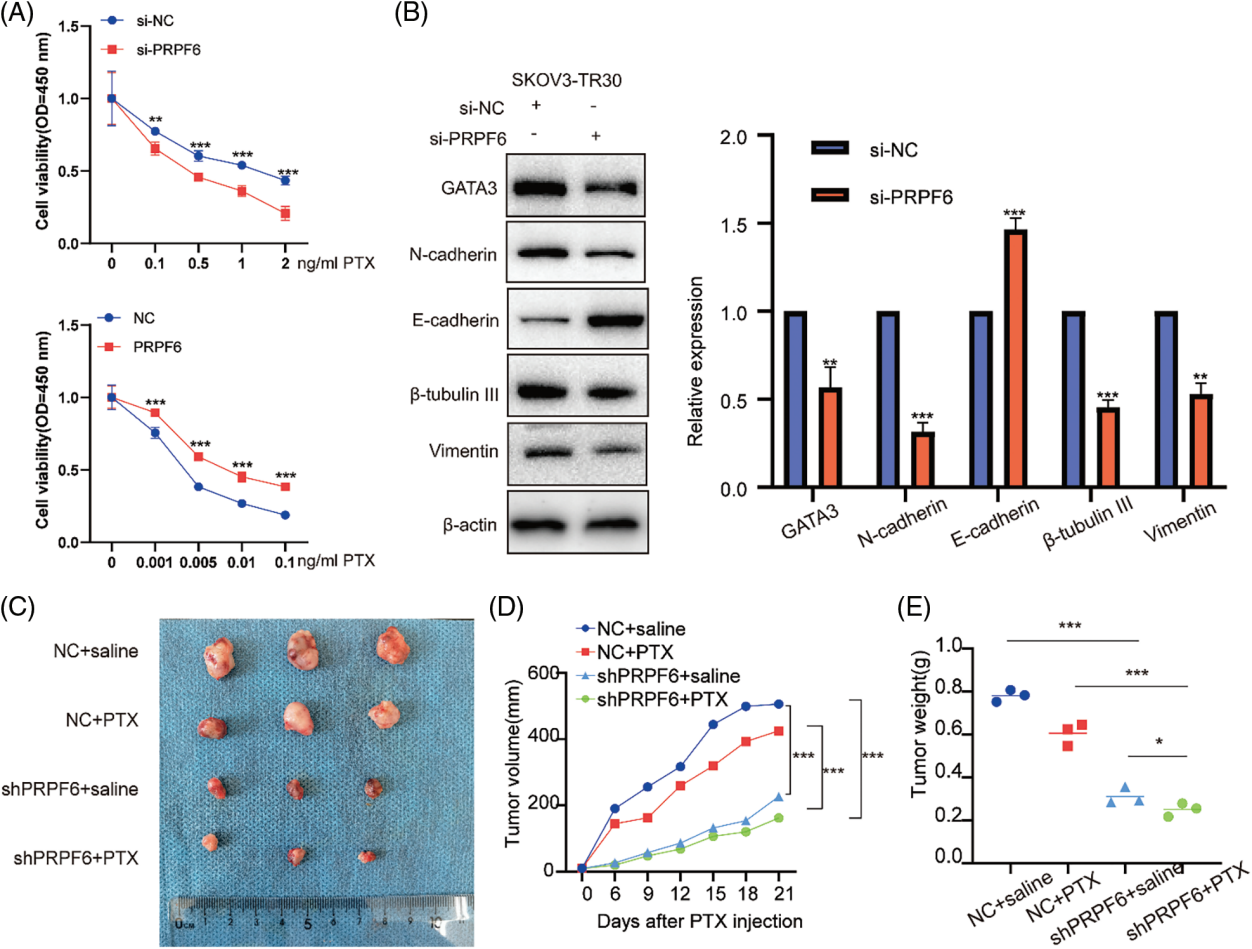
PRPF6 promoted tumorigenesis, and PTX-resistance *in vitro* and *in vivo*. (A), PTX resistance were examined by CCK8 viability assays after overexpressed PRPF6 and silencing PRPF6, (B), GATA3, N-cadherin, E-cadherin, β-tubulin III, and vimentin were detected by western blotting after silencing PRPF6. (C) SKOV3-TR30 cells were stably transfected with lentivirus containing the siPRPF6 sequence. The cells were injected in 4-week-old female BALB/cA-nu mice (N = 3/group). PTX (20 mg/kg) or saline was injected into the tumor every 3 days for 3 weeks. Images of tumors were pictured. (D) Tumor expansion was measured every 3 days (tumor volume = length × width 2/2). (E) Tumor weight was measured. *p < 0.05, **p < 0.01, ***p < 0.001 (n = 3).

Moreover, the cell viability assays after treatment with various doses of PTX indicated that si-PRPF6 inhibited PTX resistance, while PRPF6 overexpression promoted PTX resistance (Fig. 9A). *si-PRPF6* transfected in SKOV3-TR30 cells inhibited GATA3, N-cadherin, vimentin, and β-tubulin III expression and upregulated E-cadherin expression, as detected by western blotting (Fig. 9B).

To investigate function of PRPF6 *in vivo*, we constructed stable cell lines by transfecting lentiviruses containing the si-PRPF6 sequence in SKOV3-TR30 cells, and injected cells into 4-week-old female BALB/cA-nu mice (N = 3/group). Mice were divided into four groups (Fig. 9C). After a week, PTX (20 mg/kg) or saline was injected into the tumor every 3 days for 3 weeks. The results showed that knockdown of PRPF6 inhibited tumor expansion and weight (Figs. 9D and 9E). PRPF6 induced the AS of SNHG16 to upregulate GATA3 expression to promote metastasis and PTX resistance.

**Figure 10:**
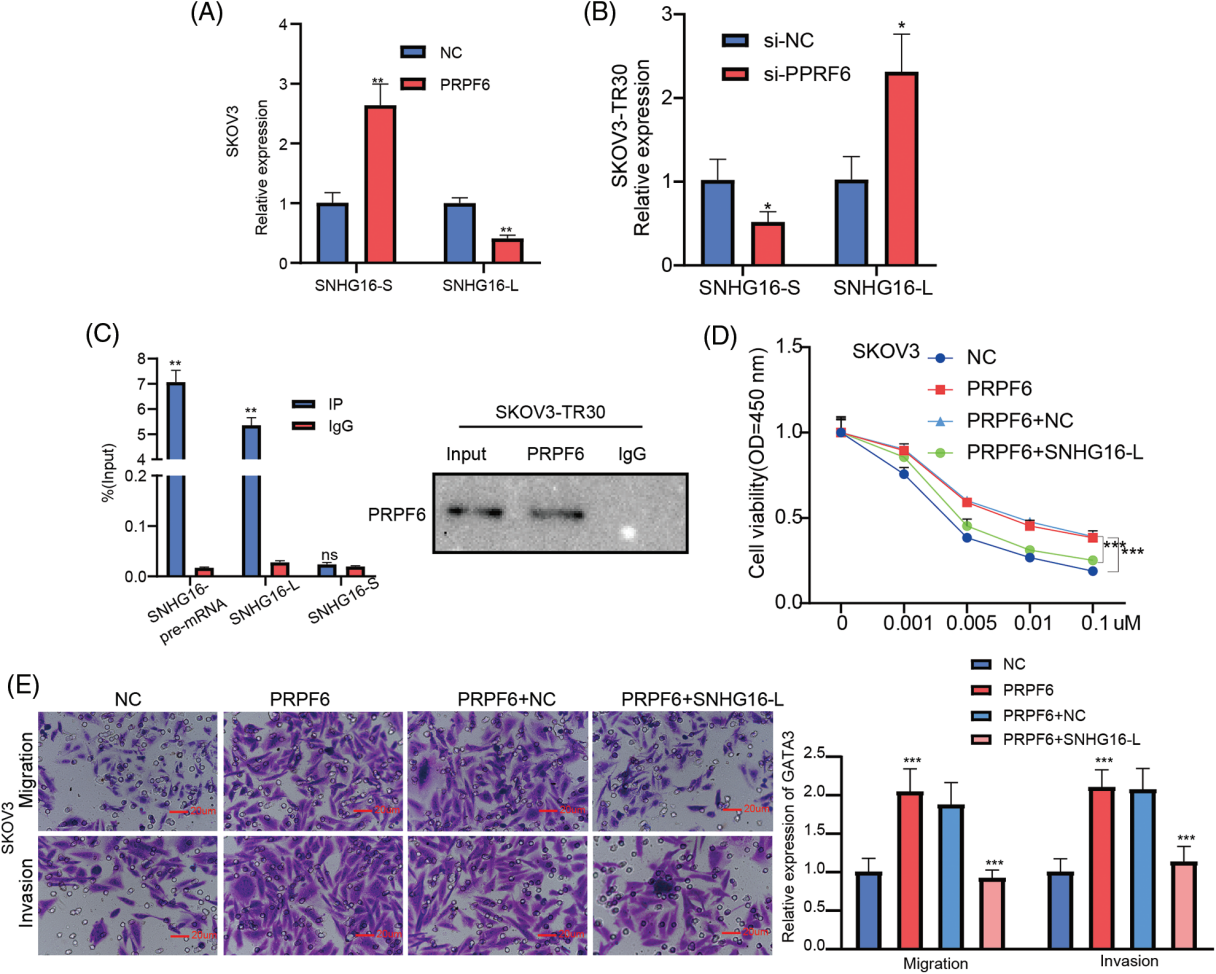
PRPF6 induced the AS of SNHG16 to promote metastasis and PTX resistance. (A&B) SNHG16-L/S expression were analyzed by RT-PCR after silencing PRPF6 in SKOV3-TR30 and overexpressing PRPF6 in SKOV3 cells. (C) RIP assay using PRPF6 antibody was carried out in SKOV3-TR30 cells, the enrichment of SNHG16 pre-mRNA, SNHG16-L and SNHG16-S was detected by RT-PCR. After transfected PRPF6 and SNHG16-L vectors in SKOV3 cells, (D) PTX resistance was examined by CCK8 vitality assays, (E) cell migration and invasion were detected by transwell assay. *p < 0.05, **p < 0.01, ***p < 0.001 (n = 3).

PRPF6 as a splicing factor, can mediate AS in various diseases [20,30]. To investigate molecular mechanism between PRPF6 and SNHG16, we first detected effects of PRPF6 differing expression on SNHG16-L/S. The results showed that overexpression of PRPF6 downregulated SNHG16-L and upregulated SNHG16-S, while silencing of PRPF6 had the opposite effects (Figs. 10A and 10B). We then conducted the RIP assay in SKOV3-TR30 cells. RT-PCR analysis showed that PRPF6 could bind to SNHG16-L and SNHG16 pre-mRNA, but not to SNHG16-S (Fig. 10C). Transwell and CCK8 assays were conducted to test the migration, invasion, PTX resistance of cells. The results indicated that the overexpression of SNHG16-L and PRPF6 weakened the promotion of PTX resistance, migration and invasion caused by PRPF6 overexpression (Figs. 10D and 10E).

**Figure 11:**
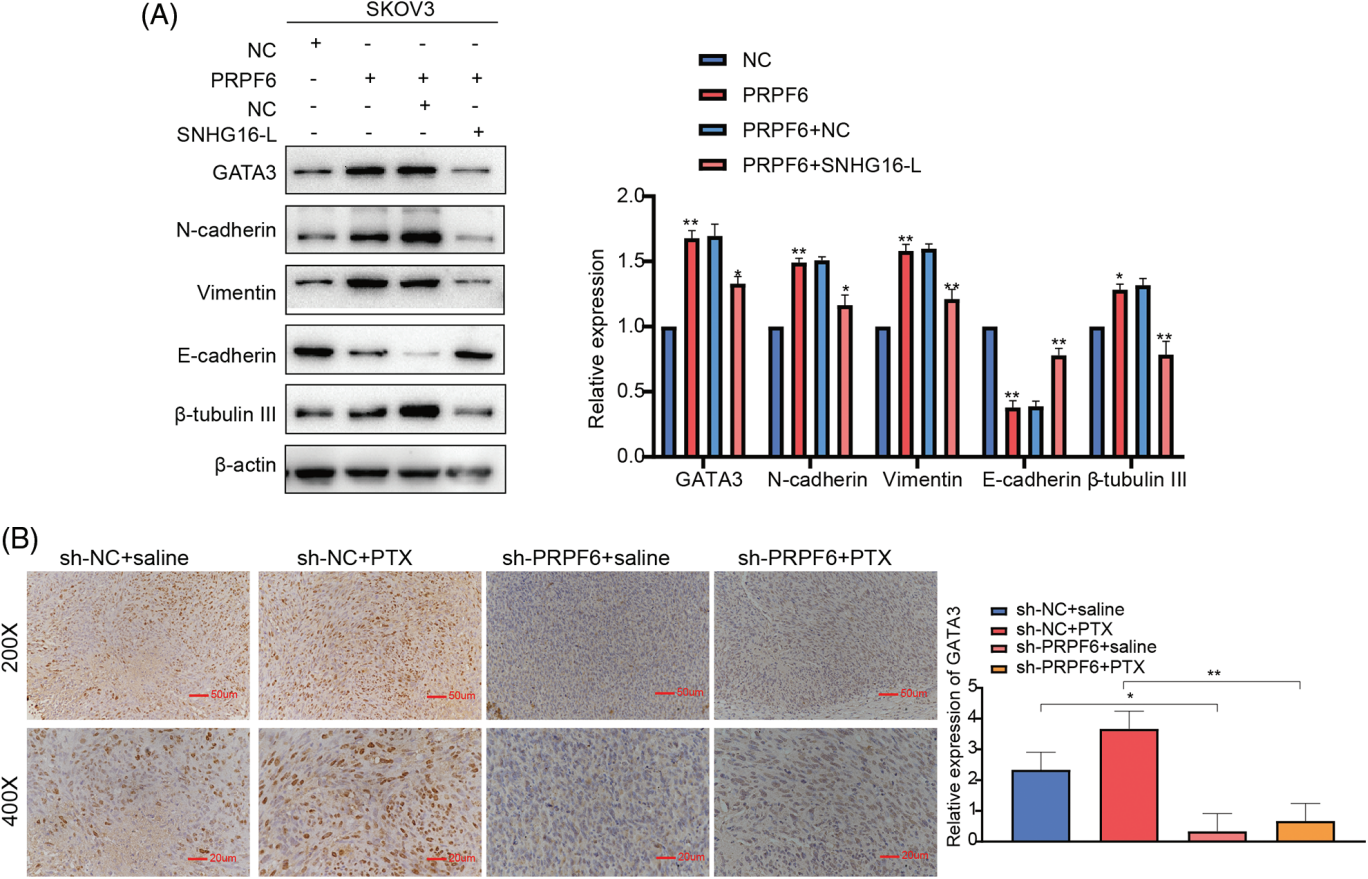
PRPF6 upregulated GATA3 expression through SNHG16-L to further induced EMT and PTX resistance. (A) GATA3, N-cadherin, E-cadherin, β-tubulin III, and vimentin expression was examined by western blotting after overexpression of PRPF6 and SNHG16-L. (B) PRPF6 and GATA3 expression in the previous xenograft tumor tissues was tested through IHC. *p < 0.05, **p < 0.01, ***p < 0.001 (n = 3).

Western blot analysis showed that overexpression of PRPF6 increased GATA3, N-cadherin, β-tubulin, III and vimentin expression and decreased E-cadherin expression, while SNHG16-L overexpression reversed this effect (Fig. 11A). To determine whether PRPF6 regulates GATA3 expression through SNHG16-L, IHC was used to detect GATA3 expression in the previous s xenograft tumor tissues. We found that knockdown of PRPF6 downregulated GATA3 *in vivo* (Fig. 11B). Taken together, these data implicate that PRPF6 promotes metastasis and PTX resistance through SNHG16/CEBPB/GATA3 axis in OC.

## Discussion

Chemo-resistance has become a key constraint to improve the cure rate of OC. PTX is the first choice of chemotherapy for advanced OC. However, the molecular mechanism of PTX resistance is still inconclusive. PTX is a mitotic inhibitor, which binds to β-tubulin III, affects the dynamic balance of microtubules, promotes the polymerization of tubulin, and blocks its depolymerization into subunits, so that cells stagnate in G2-M phase [31,32]. The expression level of β-tubulin III is positively correlated with PTX resistance of cells. And dynamic activity of tubulin in cells with high expression of β-tubulin III is the highest, which is contrary to the microtubule polymerization induced by PTX [33,34]. Thus, tumor cells can still complete mitosis even under the action of PTX, which leads to PTX resistance, which is confirmed in multiple cancers including non-small cell lung cancer, breast cancer and OC [35]. Therefore, studying the mechanism of PTX resistance is of great value for exploring effective targeted therapy and developing new drugs to reverse PTX resistance.

More than 28,000 lncRNAs have been discovered, and there are still many lncRNAs to be discovered and annotated. lncRNAs are involved in the malignant biological behavior of tumors and the regulation of chemoresistance [36]. SNHG16 has been upregulated and downregulated in multiple cancers and participates in cell proliferation, migration, invasion and EMT [37]. SNHG16 also plays an important role among gynecological tumors. SNHG16 is highly expressed in cervical cancer tissues, and is closely related to the TNM stage, tumor size, distant metastasis and survival prognosis. SNHG16 can activate Wnt/β-catenin pathway and EMT in cervical cancer [38]. In OC, there is one research showing that SNHG16 can promote cell proliferation, migration and invasion [39]. In our study, we first illustrate the different effects of SNHG16-L/S in OC. SNHG16-L overexpression inhibited tumorigenesis, EMT and PTX resistance in OC, whereas SNHG16-S induced the opposite effect. SNHG16 may therefore participate in the regulation of OC, the molecular mechanisms need to be illustrated.

EMT is the process by which epithelial cells transform into mesenchymal cells, which promotes tumor metastasis [40]. EMT shows a variety of gene expression changes, including the downregulation of epithelial genes such as E-cadherin and cytokeratin, and the upregulation of mesenchymal genes, such as N-cadherin and vimentin [41]. A decrease in E-cadherin expression is closely associated with peritoneal metastasis, and inhibits progression-free survival, and overall survival rate [42]. In addition, recent studies have shown that EMT plays a key role in chemoresistance. PTX-resistant cells can exhibit some characteristics of mesenchymal cells, including cell polarity and cell adhesion loss [43]. Genes associated with EMT, like E-cadherin, vimentin, N-cadherin, and ZEB1, are involved in regulating response of OC cells to PTX resistance [44,45]. Therefore, our study on EMT indicated SNHG16-L/S were of great significance for metastasis and PTX resistance of OC.

Increasing evidences indicate that lncRNAs can mediate transcriptional regulation by affecting the binding of transcription factors and target genes. For example, SNHG16 can recruit the SPI1 protein to promote the transcriptional activation of the PARP9 promoter in cervical cancer [27]. Mechanistically, we first report that SNHG16-L downregulates transcription activity of GATA3 through CEBPB, and inhibits migration, invasion and PTX resistance of OC. CEBPB plays a vital role in cell proliferation and tumor development. For example, CEBPB, a DNA-binding protein, can enhance the activity of the H3K79 methyltransferase, DOT1L, and regulate the methylation of H3K79 to promote cisplatin resistance in OC [46]. Moreover, CEBPB can block the transcriptional regulation of GDF15 and this effect can be inhibited by the GAS5 in OC [47]. GATA3 is highly expressed in multiple OC cell lines, and is the induction of EMT and having a poor prognosis. GATA3 promotes the arrest of the cell cycle in the G2/M phase, and induces EMT and cisplatin resistance in OC [28]. Moreover, GATA3 can interact with HIF1A to prevent its ubiquitination and proteasomal degradation, thereby promoting progression and metastasis in OC [48]. High GATA3 expression increases the ratio of p-p38MAPK/p-ERK, and promotes stemness and PTX resistance in OC [49]. Here, our findings first expose SNHG16/CEBPB/GATA3 axis in OC, which provides novel evidences for lncRNAs participating in RNA transcription.

Notably, recent studies have demonstrated that different isoforms of lncRNAs exhibit opposite functions in tumorigenesis. For example, MBNL3 regulates the AS of the lncRNA PXN-AS1 and promotes the inclusion of exon 4 to upregulate PXN in hepatocellular cancer [50]. This study provides a novel direction for future research on RBPs and lncRNAs. PRPF6, as a splicing protein, acts as an oncogene in many cancers. In this study, we first report that PRPF6 is upregulated in chemoresistant OC tissues, and closely related to advanced FIGO stages. PRPF6 promotes cell progression, metastasis, and PTX resistance in OC. PRPF6 is closely related to the advanced stage of hepatocellular cancer, which is consistent with our findings [22]. PRFP6 could therefore be a promising target for the treatment of OC. However, the relationship between PRPF6 and the survival of patients with OC still needs further exploration by expanding sample size of the cohort.

The opposing effects of SNHG16 transcripts indicated that SNHG16 might be induced by AS. AS has seven basic patterns, such as having skipped exons, retained introns, and alternate donor sites. RBPs bind to adjacent splice sites and promote the recruitment of other spliceosomes [51]. Interestingly, our study suggests that PRPF6 preferentially binds to the SNHG16 pre-mRNA and SNHG16-L, instead of SNHG16-S, indicating that PRPF6 may be possibly recruited to bind to exon 1. These data indicated that the splicing pattern of SNHG16 mediated by PRPF6 may be exon 1 skipping. However, the patterns and sites of AS remain to be investigated in-depth in future studies. In our study, we only used SKOV3 and SKOV3-TR30 cell lines. Thus, it’s necessary for us to validate the results in other OC cell lines to increase the reliability of our findings.

Collectively, our study demonstrated that PRPF6 promotes metastasis and PTX resistance via the SNHG16-L/CEBPB/GATA3 axis in OC. Our findings demonstrated the significant role of PRPF6, as a novel biomarker for OC treatment.

## Data Availability

The datasets used and/or analyzed during the current study are available from the corresponding author on reasonable request.
